# Biomarker Candidates of Habitual Food Intake in a Swedish Cohort of Pregnant and Lactating Women and Their Infants

**DOI:** 10.3390/metabo14050256

**Published:** 2024-04-29

**Authors:** Mia Stråvik, Olle Hartvigsson, Stefania Noerman, Anna Sandin, Agnes E. Wold, Malin Barman, Ann-Sofie Sandberg

**Affiliations:** 1 Food and Nutrition Science, Department of Life Sciences, Chalmers University of Technology, 412 96 Gothenburg, Swedenann-sofie.sandberg@chalmers.se (A.-S.S.); 2 Pediatrics, Department of Clinical Science, Sunderby Research Unit, Umeå University, 901 87 Umeå, Sweden; 3 Department of Infectious Diseases, Institute of Biomedicine, Sahlgrenska Academy, University of Gothenburg, 413 90 Gothenburg, Sweden

**Keywords:** dietary biomarkers, metabolomics, pregnancy, lactation, infants, lutein, proline betaine

## Abstract

Circulating food metabolites could improve dietary assessments by complementing traditional methods. Here, biomarker candidates of food intake were identified in plasma samples from pregnancy (gestational week 29, N = 579), delivery (mothers, N = 532; infants, N = 348), and four months postpartum (mothers, N = 477; breastfed infants, N = 193) and associated to food intake assessed with semi-quantitative food frequency questionnaires. Families from the Swedish birth cohort Nutritional impact on Immunological maturation during Childhood in relation to the Environment (NICE) were included. Samples were analyzed using untargeted liquid chromatography–mass spectrometry (LC-MS)-based metabolomics. Both exposure and outcome were standardized, and relationships were investigated using a linear regression analysis. The intake of fruits and berries and fruit juice were both positively related to proline betaine levels during pregnancy (fruits and berries, β = 0.23, FDR < 0.001; fruit juice, β = 0.27, FDR < 0.001), at delivery (fruit juice, infants: β = 0.19, FDR = 0.028), and postpartum (fruits and berries, mothers: β = 0.27, FDR < 0.001, infants: β = 0.29, FDR < 0.001; fruit juice, mothers: β = 0.37, FDR < 0.001). Lutein levels were positively related to vegetable intake during pregnancy (β = 0.23, FDR < 0.001) and delivery (mothers: β = 0.24, FDR < 0.001; newborns: β = 0.18, FDR = 0.014) and CMPF with fatty fish intake postpartum (mothers: β = 0.20, FDR < 0.001). No clear relationships were observed with the expected food sources of the remaining metabolites (acetylcarnitine, choline, indole-3-lactic acid, pipecolic acid). Our study suggests that plasma lutein could be useful as a more general food group intake biomarker for vegetables and fruits during pregnancy and delivery. Also, our results suggest the application of proline betaine as an intake biomarker of citrus fruit during gestation and lactation.

## 1. Introduction

Dietary assessments have well-documented flaws since many methods rely on human memory and honesty [1]. Indeed, the most common methods to measure dietary intake, such as 24 h recalls, food frequency questionnaires, and food diaries, are all subjective and prone to recall bias and inaccurate estimations of portion sizes [1,2]. One way to overcome the subjectivity of estimating one’s food intake is to also measure food metabolites in the blood. Metabolites that reflect the consumption of a specific food can be considered a food intake biomarker [3] and can complement traditional dietary assessment methods.

Some food intake biomarkers are validated and well established, such as proline betaine for citrus fruit [4] and docosahexaenoic acid (DHA) for fatty fish [5]. In contrast, there are several candidate biomarkers that are often mentioned in the literature (e.g., pentadecanoic acid for dairy product intake) but remain to be fully validated and, hence, need further investigation [6,7]. The criteria for biomarker validation (e.g., plausibility, dose–response, time–response) were suggested in 2018 [8] and were recently adapted to be more applicable to epidemiological studies by adding an association with habitual food intake into the validation criteria [6].

Although potential dietary biomarkers have emerged from previous studies, the validity of using these markers under different physiological conditions and in a non-controlled setting remains to be elucidated. Specifically, the biomarker discoveries during pregnancy or lactation, during which a woman should provide both herself and a growing fetus and infant with nutrients [9], have rarely been explored. Not only may the metabolism be changed in the pregnant state, but some nutrients are also actively transported across the placenta to meet the needs of the fetus [10]. During lactation, the woman is depleted of nutrients by producing roughly a liter of breast milk daily. This can affect the turnover of metabolites in women’s plasma, including potential food intake biomarkers [11,12]. Hence, previously validated biomarkers of food intake may not be suitable for use in pregnant and lactating women. On the other hand, having an objective and validated panel of dietary biomarkers for this group is very important, as inadequate nutrition during pregnancy can have major consequences for the health and development of the fetus/child [9].

Therefore, this study aimed to investigate a set panel of candidate food intake biomarkers using untargeted metabolomic data from liquid chromatography coupled with tandem mass spectrometry (LC-MSMS) on plasma samples taken from pregnant and lactating women and their infants in the Swedish birth cohort Nutritional impact on Immunological maturation during Childhood in relation to the Environment (NICE). The relative intensities of these plasma metabolites were associated with maternal self-reported food intake assessed with a semi-quantitative food frequency questionnaire at corresponding time points.

## 2. Materials and Methods

### 2.1. Study Population

Mothers and infants were selected from the Swedish birth cohort NICE (N = 655 families) based on the availability of metabolomic data and dietary data, as shown in Table 1. A total of 579 women, 348 newborns, and 193 infants were studied here. Pregnant women were recruited around gestational week 18 during a visit to the local maternity clinic for a routine ultrasound. Inclusion criteria included a planned childbirth at Sunderby Hospital, Northern Sweden, and the ability to communicate in Swedish. Final inclusion was performed at delivery during the recruitment period from February 2015 to March 2018. Written informed consent was obtained from all expecting parents concerning themselves and their expected child. The study was conducted in accordance with the Helsinki Declaration and approved by the Regional Ethical Review Board in Umeå, Sweden (2013/18-31M, 2015-71-32). Blood sampling occurred during pregnancy, delivery, lactation, and early childhood, and food intake and lifestyle parameters were investigated using different questionnaires. The study protocol has been published elsewhere [13].

### 2.2. Dietary Assessment

Diet was assessed with a repeated web-based semi-quantitative food frequency questionnaire (Meal-Q) in gestational week 34 (reflecting intake between gestational weeks 30–34), at one month postpartum (reflecting intake between childbirth and one month), and at four months postpartum (reflecting intake between three and four months after delivery). The collection and design of the questionnaire have previously been described in detail [14,15]. Briefly, intake in grams per day was estimated based on reported intake frequency (1–3 times/month to ≥5 times/day) and reported portion size (images of five different portion sizes). Food groups were created by summarizing the intake in grams/day of all relevant food items [14]. The response rates for the food frequency questionnaires were 94%, 93%, and 93%, respectively.

### 2.3. Plasma samples

In gestational week 29 (5th–95th percentiles: 27–32), venous blood from the cubital vein was collected in 10 mL EDTA tubes (Becton, Dickinson and Company, Franklin Lakes, NJ, USA). The samples were drawn at the local maternity health clinics and stored at 4 °C until transported cold to the hospital laboratory the same or, at the latest, the following workday.

During childbirth (usually at arrival to the delivery ward), venous blood from the mother’s cubital vein was collected in 10 mL EDTA tubes (Becton, Dickinson and Company, Franklin Lakes, NJ, USA). Immediately after the child was born, blood was also collected from the clamped umbilical cord by severing it and squeezing out the blood into 6 mL EDTA tubes (Becton, Dickinson and Company, Franklin Lakes, NJ, USA). The samples from this time point were stored at 4 °C until transported to the hospital laboratory the same day (if deliveries occurred during working hours) or the following workday (if deliveries occurred outside working hours). Samples could be stored uncentrifuged for up to three days. More detailed information on time from sampling until freezing has been presented elsewhere [16].

Four months after birth, venous blood samples from the dorsal side of the hand were collected from the infants using 3 mL EDTA tubes (Becton, Dickinson and Company, Plymouth, UK) and from the cubital vein of the mothers using 10 mL EDTA tubes (Becton, Dickinson and Company, Franklin Lakes, NJ, USA).

All samples were temporarily stored at 4 °C until centrifuged at the hospital laboratory at 2.400 rpm for five minutes (Hettich Rotina 420, Hettich Lab Technology, Tuttlingen, Germany) to separate the plasma before freezing at −80 °C for long-term storage.

### 2.4. Metabolomics

**Figure 1:**
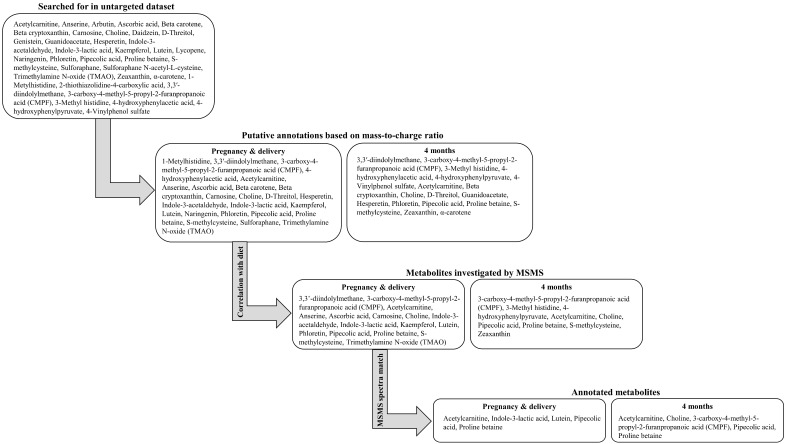
Method flowchart for the selection of metabolites to be included.

Plasma samples from pregnancy (mothers), delivery (mothers and infants), and four months postpartum (mothers and infants) were analyzed with untargeted liquid chromatography–mass spectrometry (LC-MS). Instrument settings have previously been described in detail [16]. To note, samples from pregnancy and delivery were analyzed separately from the samples collected four months postpartum using two different instruments. Features from the untargeted analysis (not yet annotated, referred to by mass-to-charge ratio and retention time) were selected from a literature survey and communication performed as part of the EU project food phytochemicals matter for cardiometabolic health (FOODPHYT) under the joint programming initiative a Healthy Diet for a Healthy Life (not published) [17]. The features with putative identifications based on mass-to-charge ratio (N = 63 in samples from pregnancy and delivery and N = 29 in samples from four months postpartum) were correlated to a wide range of food items (N = 61). Features of interest for further investigation were selected based on plausibility to be found in or related to the intake of the correlated food item. Maternal samples with the highest intensity of these features were reanalyzed with mass spectrometric fragmentation (MSMS) to obtain product ion spectra for compound annotation (Figure 1 and Table S1).

#### 2.4.1. Annotation of Metabolites

The annotation procedure was conducted after extracting the raw data from the tandem mass spectrometry. An in-house developed R-based Shiny app (OCEAN) was used to match the MSMS spectra with library spectra from MS-DIAL spectral libraries (~350,000 spectra of ~25,000 compounds) [18]. Metabolites were manually compared against the HMDB spectral library [19]. All metabolites identified with this approach were classified as level 2 identification (i.e., probable structure from library spectrum match and diagnostic evidence) as suggested by Schymanski et al. [20]. The preselected features that could not be annotated were classified as level 5 (i.e., confidence solely based on exact mass) and considered too uncertain to investigate further.

### 2.5. Statistical Analyses

Data were analyzed with R version 3.6.2 (R Foundation for Statistical Computing, Vienna, Austria) software packages. The relative intensity of the annotated features (i.e., MS data, continuous) was investigated in relation to reported food intake (i.e., food frequency questionnaire data, grams/day) using linear regression models. To explore the relationships between the relative intensities of metabolites in mothers and their children (i.e., not involving diet), Spearman correlation analysis was used.

Three different levels of adjustment were used in the regression analyses: (1) without any adjustment for confounders; (2) adjusted for age, education, and parity; and (3) adjusted for age, education, parity, and total energy intake. Both exposures (food intake) and outcomes (metabolites) were centered and scaled before inserted into the regression models. The regression coefficients for the main model (adjusted for age, education, and parity) were visualized using heatmaps. A directed acyclic graph was used in the process of choosing confounders and main model (Figure S1). Exact numbers from all regression models and the Spearman correlations are presented in Supplementary Materials.

All *p*-values were adjusted for the false discovery rate (FDR) using the Benjamini–Hochberg procedure. Adjusted *p*-values below 0.05 were considered significant for all tests.

## 3. Results

### 3.1. Annotation of Metabolites

In total, 37 potential features of interest were selected for the MSMS fragmentation analysis. These features were expected, solely based on mass-to-charge ratio, to correspond to 19 unique metabolites represented in a wide range of foods: specific food items (e.g., kaempferol in apples) and more broad food groups (e.g., carnosine in animal tissue).

Table 2 displays annotated features with mass-to-charge ratio and retention time, and Figures S2–S8 show the best spectra match for level 2 annotations. The annotated features included acetylcarnitine, choline, indole-3-lactic acid, lutein, pipecolic acid, proline betaine, and 3-carboxy-4-methyl-5-propyl-2-furan-propanoic acid (CMPF). Acetylcarnitine, pipecolic acid, and proline betaine were identified in samples from all three time points (Table 2).

### 3.2. Relationship between Metabolites and Reported Food Intake

The relative intensities of the seven identified candidate food intake biomarkers in maternal and infant plasma were related to the reported intake of various food items at the corresponding time points using a linear regression analysis. All dietary variables and metabolites underwent mean centering and scaling, and the *p*-values are presented adjusted for the false discovery rate (FDR).

**Figure 2:**
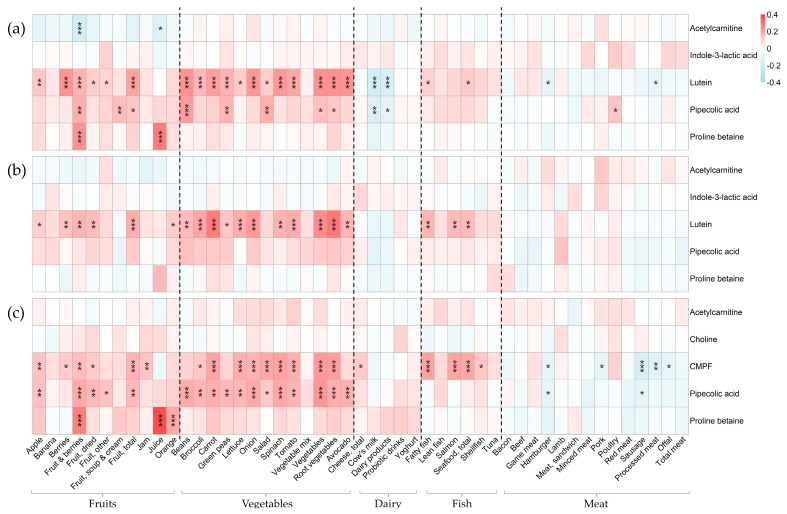
Linear regression between annotated metabolites in maternal plasma and food intake during (**a**) pregnancy, (**b**) at delivery, and (**c**) at four months postpartum. The models were adjusted for age, education, and parity. All metabolites and food variables were standardized to have the mean of zero and standard deviation of one. The magnitude of each regression coefficient is denoted by color, whereby red indicates a positive association and blue represents a negative association. Darker shades of these two colors indicate stronger associations. *p*-values were FDR adjusted, and significant associations are denoted as * *p* < 0.05, ** *p* < 0.01, and *** *p* < 0.001.

**Figure 3:**
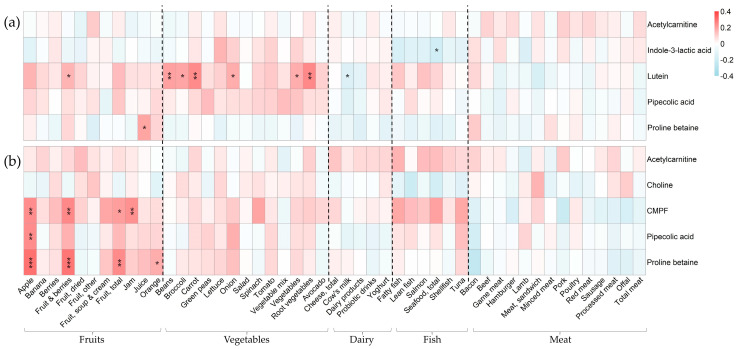
Linear regression between annotated metabolites in infant plasma and maternal food intake during (**a**) delivery and (**b**) at four months postpartum. The models were adjusted for age, education, and parity. All metabolites and food variables were standardized to have a mean of zero and standard deviation of one. The magnitude of each regression coefficient is denoted by color, whereby red indicates a positive association and blue represents a negative association. Darker shades of these two colors indicate stronger associations. *p*-values were FDR adjusted, and significant associations are denoted as * *p* < 0.05, ** *p* < 0.01, and *** *p* < 0.001.

Figure 2 shows the relationship between food intake biomarker levels in maternal plasma from (a) pregnancy, (b) delivery, and (c) four months postpartum and the corresponding food intake. Figure 3 shows the relationship between infant plasma metabolites during (a) delivery and (b) at four months postpartum and maternal food intake at the corresponding time points. The results from these analyses are presented in separate sections below, one for each identified candidate biomarker. More detailed results (β coefficients, unadjusted *p*-values, and adjusted *p*-values) can be found in the Supplementary Materials.

#### 3.2.1. Proline Betaine and Intake of Citrus Fruit

Proline betaine, a non-essential permethylated amino acid, is abundant in citrus fruit and a validated intake biomarker [4,23]. Here, we found that proline betaine in women’s plasma was positively related to the reported intake of fruit juice (commonly orange juice) during pregnancy (β = 0.27, FDR < 0.001) (Figure 2a) and at four months postpartum (β = 0.37, FDR < 0.001) (Figure 2c). Proline betaine also correlated with the reported intake of fruits and berries during pregnancy (β = 0.23, FDR < 0.001) and postpartum (β = 0.27, FDR < 0.001) and, more specifically, also with the reported intake of oranges postpartum (as a whole fruit) (β = 0.18, FDR < 0.001).

Regarding the infants’ plasma levels of proline betaine, a positive association was found with maternal fruit juice intake at delivery (β = 0.19, FDR = 0.028) (Figure 3a). During breastfeeding, the infants’ plasma levels were significantly related to the maternal intake of apple (β = 0.30, FDR < 0.001), fruits and berries (β = 0.29, FDR < 0.001), total fruit (β = 0.24, FDR = 0.002), and oranges (β = 0.18, FDR = 0.016) (Figure 3b).

#### 3.2.2. Lutein and Intake of Fruit and Vegetables

Lutein is a carotenoid synthesized solely by plants, leading to high concentrations of lutein in such food sources (e.g., spinach and other green leafy vegetables). Indeed, lutein levels in the plasma of women during pregnancy and delivery were positively associated with the reported intake of a wide range of vegetables and fruits (Figure 2a,b). During pregnancy, the five strongest associations between plasma lutein and food intake were seen for (ranging from highest to lowest, β ≈ 0.23 for all) vegetables, beans, root vegetables, avocados, and berries (Figure 2a). At the time of delivery, similar relationships were found for the mentioned vegetables and fruits, but the top five associations were seen for (ranging from highest to lowest, β = 0.28–0.19) root vegetables, carrots, vegetables, onions, and broccoli (Figure 2b).

Lutein was also present in infant plasma, and the cord blood levels partially reflected the maternal intake of vegetables and fruits (Figure 3). Significant associations were seen for lutein in umbilical cord blood and the maternal intake of root vegetables (β = 0.25), carrots (β = 0.24), beans (β = 0.20), broccoli (β = 0.19), onion (β = 0.18), vegetables (β = 0.18), and fruits and berries (β = 0.16). Lutein was not identified in either maternal or infant samples from four months postpartum.

#### 3.2.3. Pipecolic Acid and Intake of Plant-Based Food

Pipecolic acid, a metabolite of the essential amino acid lysine, might originate from endogenous sources (e.g., gut microbiota metabolism) or exogenous sources (e.g., the intake of beans). Several associations were found between diet and plasma levels. Pipecolic acid in plasma during pregnancy was most strongly associated with the intake of beans (β = 0.21, FDR < 0.001), salad (β = 0.17, FDR = 0.001), fruits and berries (β = 0.17, FDR = 0.001), and green peas (β = 0.16, FDR = 0.004), but significant associations were also found for (β ranging from 0.14 to 0.12) fruit soup and fruit cream, vegetables, total fruit, poultry, and root vegetables (Figure 2a).

At the time of delivery, maternal food intake did not relate to the pipecolic acid levels in neither her own plasma nor her infant’s plasma (Figure 2b and Figure 3a).

At four months postpartum, however, several associations were once again found between the reported intake of plant-based food and pipecolic acid levels in maternal plasma. At this timepoint, the strongest associations were found for the intake of vegetables (β = 0.22, FDR < 0.001), spinach (β = 0.18, FDR = 0.001), fruits and berries (β = 0.18, FDR = 0.001), and beans (β = 0.18, FDR < 0.001), but significant associations were also found for (β ranging from 0.18 to 0.11) onions, root vegetables, avocados, carrots, tomatoes, broccoli, green peas, dried fruits, lettuce, total fruits, apple, other fruits, and salad (Figure 2c).

Regarding the infants’ plasma levels at four months, a positive relationship was found with the maternal intake of apples (β = 0.24, FDR = 0.002) (Figure 3b).

#### 3.2.4. CMPF and Intake of Seafood

3-carboxy-4-methyl-5-propyl-2-furan-propanoic acid (CMPF), a metabolite of furan fatty acids, is a biomarker of fish intake. The metabolite was identified in samples from four months postpartum. As can be seen in Figure 2c, the relative intensity of CMPF in maternal plasma samples was positively related to the intake of fatty fish (β = 0.20, FDR < 0.001), including salmon in particular (β = 0.19, FDR < 0.001), and with the total intake of seafood (β = 0.18, FDR < 0.001) and shellfish (β = 0.12, FDR = 0.021).

Regarding the CMPF levels of the infants and their relationship to maternal food intake, none of the expected food sources (i.e., seafood) were found significantly associated with the plasma levels (Figure 3b).

#### 3.2.5. Choline and Intake of Animal Products

Choline, a nutritional component found in a wide range of foods (e.g., offal, beans, nuts) [24] but with the major contribution coming from animal products (e.g., chicken, egg, and cow’s milk) [25], was identified in plasma samples from four months postpartum. Neither maternal nor infant plasma levels of choline were associated with any of the expected food sources (Figure 2c and Figure 3b).

#### 3.2.6. Acetylcarnitine and Intake of Animal Products

Acetylcarnitine levels in plasma were identified at all timepoints. At none of the timepoints were the levels related to the intake of any expected food source (i.e., animal products) (Figure 2 and Figure 3).

#### 3.2.7. Indole-3-Lactic Acid and Intake of Tryptophan-Rich Food

The levels of indole-3-lactic acid, which is endogenously produced after the consumption of tryptophan-rich food (e.g., dairy products, meat, and oats), did not associate with any of the expected food sources during pregnancy (Figure 2a) or delivery (Figure 2b and Figure 3a). The metabolite could not be identified in samples from four months postpartum.

### 3.3. Maternal Metabolites in Relation to Offspring Metabolites

**Figure 4:**
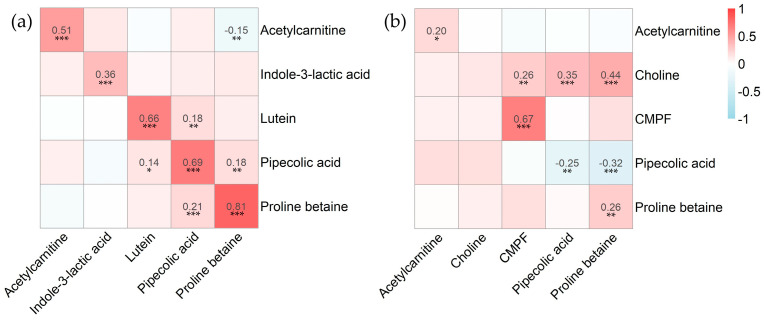
Spearman correlations between annotated metabolites in infant plasma (*x*-axis) and maternal plasma (*y*-axis) during (**a**) delivery and (**b**) at four months postpartum. *p*-values were FDR adjusted, and significant associations are denoted as * *p* < 0.05, ** *p* < 0.01, and *** *p* < 0.001.

As previously described, blood samples were obtained from infants at delivery and four months postpartum. At the latter time point, only breastfed infants were included in the statistical analyses. As pointed out above, there were some positive relationships between plasma metabolites in umbilical cord blood (lutein and proline betaine) and the maternal intake of fruits and vegetables, indicating that these molecules could have passed over already in utero (Figure 3a). The relative intensities of these specific metabolites correlated more strongly between the mother’s and the child’s plasma levels than those metabolites in umbilical cord plasma which did not correlate with the maternal diet (indole-3-lactic acid and acetylcarnitine), with the exception of pipecolic acid (Figure 4a).

At four months postpartum, positive associations were noted between infant plasma CMPF, proline betaine, and pipecolic acid levels and the maternal intake of fish and fruits (Figure 3b). Interestingly, CMPF and proline betaine correlated more strongly between mothers and infants than those in infant plasma without any association with maternal diet, indicating transportation via breast milk, whilst maternal and infant pipecolic acid levels were negatively associated with each other (Figure 4b). To note, the median age for when these children were first introduced to fish in their own diet was six months and to fruits and fruit juice at four months of age (between four and five months, i.e., after blood sampling).

## 4. Discussion

The present study aimed to investigate a panel of candidate food intake biomarkers in a cohort of women during pregnancy, at delivery, and at four months postpartum. These markers were also analyzed in breastfed infants’ blood, sampled at delivery and four months postpartum, suggesting that the metabolites could pass across the placenta or be transferred via breast milk. The results indicate that circulating levels of proline betaine can be useful for reflecting habitual citrus fruit intake collected with a semi-quantitative food frequency questionnaire during pregnancy and the first months postpartum. In addition, plasma levels of lutein seem related to the intake of a wide range of vegetables and fruits during pregnancy and at delivery. This could indicate its usefulness as a broader, less specific food intake biomarker for a plant-based diet.

The most pronounced relationship was seen for circulating proline betaine and self-reported citrus fruit intake during pregnancy and at four months postpartum. Since humans cannot synthesize the metabolite, it must be consumed in the diet to be measurable in plasma. Our results align with previous studies, which have demonstrated its usefulness as an intake biomarker for citrus fruit when measured in urine [4], although the within-person variation in urine seems high among pregnant women [26].

Lutein is available in various vegetables and fruits [27], and in our study, plasma levels of the metabolite during pregnancy and at delivery and in the umbilical cord were associated with the reported intake of vegetables and fruits. The strongest associations were found for vegetables in general and root vegetables. The variable for root vegetables included carrots, beetroot, parsnip, Swedish turnip, and celeriac (celery root), and the association was mainly driven by the intake of carrots, which has previously been shown to be a rich source of lutein [28].

Plasma levels of CMPF were indeed associated with the expected food sources (i.e., seafood) in maternal plasma four months postpartum. However, several unexpected associations were also seen. In addition to seafood, the metabolite was positively associated with a wide range of fruits and vegetables. However, a causal effect of the intake of fruits and vegetables on CMPF plasma levels seems highly unlikely. To the best of our knowledge, fruits and vegetables are neither rich sources of furan fatty acids nor have such associations been reported in the previous literature. This association was likely due to fish and vegetable intake being positively correlated in this group of women (e.g., apple and fatty fish: rho = 0.20, *p* < 0.001). Along the same lines, some biomarkers were negatively associated with the intake of some foods (e.g., acetylcarnitine with fruits and berries and lutein with cow’s milk), which we suggest depends on negative associations between certain food groups (e.g., spinach and cow’s milk: rho = −0.17, *p* < 0.001) [14,15].

Although CMPF could not be detected during pregnancy and delivery, we have previously found positive associations between fish intake during pregnancy and proportions of n-3 PUFAs in maternal phospholipids at delivery [29] as well as with mercury and arsenic concentrations in maternal erythrocytes during pregnancy [30], indicating that they could better reflect seafood intake at this stage of life.

The lack of association between plasma levels of choline and maternal food intake may be explained by the fact that food is not the only source of choline; it can also be endogenously produced in the liver [31]. Further, both the fetus and the infant have a high demand for choline due to its role in brain development as a vital constituent in membrane phospholipids. Therefore, choline transportation via the placenta is prioritized over meeting the maternal need [32,33], and choline transportation via breast milk during the first months is high and estimated to be 120 mg/day. However, this estimation is based on several assumptions [34]. Hence, the use of plasma levels of choline as an intake biomarker of animal products can be questioned.

Dairy products (source of pipecolic acid) and pipecolic acid were not related to each other, which could be explained by a larger impact on plasma levels by the lysine-rich food rather than the intake of pipecolic acid from dairy products. Previous research has shown that levels of pipecolic acid in plasma are more affected by lysine metabolism and, hence, the intake of lysine-rich foods than by the intake of the metabolite *per se* [35]. The suitability of using pipecolic acid as a marker of dairy products can be questioned, and we have, in a previous study within the same cohort, demonstrated that pentadecanoic acid (15:0) measured in breast milk and erythrocytes could be a more useful intake biomarker of cow’s milk [14].

As reviewed elsewhere, indole-3-lactic acid has been suggested as a fermentation-dependent candidate biomarker for soy products and yoghurt [36]. We could not identify any associations to dietary intake in our study. It is possible that the food frequency questionnaires did not capture the level of details needed for these products (i.e., fermentation information) or, simply, that the plasma levels are affected by several different sources. For instance, the impact of tryptophan-rich food on indole levels in the blood is mediated by the gut microbiota, which could be affected by dietary fiber intake [37,38].

Even though there are several advantages to using objective biomarkers to measure food intake, there are also many difficulties. For example, many candidate food intake biomarkers are not exclusively found in one food item but in several food products (e.g., acetylcarnitine, choline, indole-3-lactic acid, and lutein). Also, some candidate biomarkers are endogenously produced in the body from the metabolism of other compounds (e.g., tryptophan metabolism resulting in the production of indole-3-lactic acid) [39]. Further, the feasibility of using different food intake biomarkers during pregnancy (i.e., at a time with extensive physiological changes, such as increased plasma volume [40] and fetal demands) can be questioned, which has previously been brought up [41]. For instance, the plasma concentrations of choline have been found to steadily increase throughout the pregnancy, explained partly by storage depletion and increased synthesis to ensure fetal needs [42]. Hence, direct food intake containing these compounds is not the sole impactor of the plasma levels [31,35,39].

Our study design did not allow for the exact quantification of either metabolite concentrations or food intake. Due to the exploratory and semi-quantitative nature of untargeted metabolomics, with both food intake and metabolite levels being standardized, the regression coefficients indicate directionality rather than an absolute estimate of changes in metabolite concentrations. While previous studies have identified biomarkers of food intake, few have specifically explored their reliability across different physiological states, such as pregnancy, delivery, and lactation.

One cohort of pregnant women in Norway found a moderate association between plasma concentrations of lutein and FFQ-reported intake of fruits and vegetables [43]. Another study pooled the results from three different pregnancy cohorts and found moderate associations between proline betaine in serum and fruit juice intake [44]. Since the analytical approach was different between these studies and ours (e.g., correlation versus regression, metabolite concentrations versus relative intensities), the strength of associations cannot be compared but the direction of associations seem confirmatory. To the best of our knowledge, this study is the first to demonstrate that proline betaine levels in plasma remain consistently reliable indicators throughout pregnancy and into the postpartum period, an insight not previously reported.

The fact that some of the metabolites (e.g., CMPF and lutein) were not possible to measure at all time points can have several explanations, which must be acknowledged. The samples from four months were analyzed using another LC-MS system, and the generalizability of the data obtained with different mass spectrometers is a known issue within metabolomics [45]. In addition, the blood samples at four months were collected at the study center and centrifuged, aliquoted, and frozen in close conjunction with the sampling, while the samples from pregnancy were collected at different maternity health clinics (i.e., various routines and time until freezing). The samples at delivery were all collected at the hospital, and the time until the sample was centrifuged and frozen varied depending on the day the delivery occurred (i.e., weekday or weekend) and at what time (i.e., outside office hours).

Our longitudinal approach, tracking biomarker levels from pregnancy through to the postpartum period, the cross-sectional analysis at each stage, and the use of a repeated, semi-quantitative food frequency questionnaire with a high response rate are some of the strengths with this study. Also, the use of LC-MS enabled the detection of a broader range of metabolites compared to GC-MS and NMR [46]. Further, an extensive collection of plasma samples was performed from mothers and their infants at repeated time points, including plasma from the umbilical cord. However, one must keep in mind that the blood samples were taken in gestational week 29, at delivery, and at the four-month follow-up, while the food intake was assessed in gestational week 34, at one month postpartum, and at four months postpartum, reflecting the intake one month back in time. It is important to remember that some metabolites may have fast turnover in plasma and might not have been captured well in this study. However, the risk of participants changing their diet between blood sampling and dietary assessment is low since dietary habits typically take more than a few weeks to change [47]. Hence, variations are more likely attributed to day-to-day variations in consumed amounts, which might dilute the associations rather than cause an actual change in habitual food intake. The associations found here do not indicate any dose–response relationship. Such relationships remain to be investigated in future studies under more controlled settings.

## 5. Conclusions

Our study suggests that proline betaine in plasma can be used as an intake biomarker of citrus fruit during pregnancy and the first months postpartum. In addition, plasma levels of lutein were associated with the intake of a wide range of vegetables and fruits, which could strengthen its usefulness as a more general food group intake biomarker. However, given the nature of this study, the results must be interpreted as exploratory, and no conclusions regarding dose–response can be drawn.

## Figures and Tables

**Table 1:** Samples included in the study.

	Pregnancy ^1^	Delivery ^2^		Lactation ^3^	
	Mothers	Mothers	Infants	Mothers	Infants
Metabolomic data	611	562	370	497	240
Metabolomics + FFQ	579	532	348	477	239
Breastfed					193 ^4^
Analyzed samples	579	532	348	477	193

^1^ Gestational week 29 (blood) and gestational week 30–34 (diet). ^2^ At the delivery ward (blood) and the first month postpartum (diet). ^3^ Four months postpartum (blood) and three to four months postpartum (diet). ^4^ N = 35 infants were excluded due to not being breastfed and another N = 11 infants because of lack of breastfeeding data.

**Table 2:** Annotated features in maternal and infant blood and confidence level.

Mass-to-Charge Ratio	RT (Min)	Ionization	Putative Annotation	Confidence ^1^	Adduct	Fragments (Relative Intensity)	HMDB ID	Reference
**Pregnancy and delivery ^2^**								
130.086	0.89	Positive	Pipecolic acid	Level 2	M + H	40 V: 56.05 (100), 60.987 (75), 84.081 (48), 69.056 (45), 55.934 (44), 81.938 (27), 42.033 (24)	HMDB0000070	[19]
144.101	0.78	Positive	Proline betaine	Level 2	M + H	144.103 (100), 58.065 (38), 84.08 (33), 98.097 (27), 70.065 (15), 102.056 (8), 72.081 (7)	HMDB0004827	[21]
204.067	3.71	Negative	Indole-3-lactic acid	Level 2	M − H	158.06 (100), 116.051 (58), 128.051 (54), 142.068 (51), 130.066 (36), 186.056 (24), 103.055 (20)	HMDB0000671	[19]
204.123	0.80	Positive	Acetylcarnitine	Level 2	M + H	85.029 (100), 204.124 (77), 60.081 (29), 145.049 (24), 187.018 (5), 122.061 (4), 144.103 (3)	HMDB0000201	[19]
568.428	7.81	Positive	Lutein	Level 2	M+	568.428 (100), 338.261 (93), 476.364 (59), 173.134 (38), 89.06 (36), 211.149 (33), 138.103 (32)	HMDB0003233	[19]
**4 months postpartum**								
104.107	0.68	Positive	Choline	Level 2	M + H	60.081 (100), 45.034 (45), 58.065 (31), 104.107 (27), 45.057 (12), 59.073 (8), 44.05 (7)	HMDB0000097	[22]
130.086	0.93	Positive	Pipecolic acid	Level 2	M + H	20V: 84.081 (100), 130.087 (31), 68.994 (25), 84.045 (23), 88.982 (17), 67.997 (8), 130.05 (5)	HMDB0000070	[19]
144.102	0.77	Positive	Proline betaine	Level 2	M + H	144.103 (100), 58.065 (38), 84.08 (33), 98.097 (27), 70.065 (15), 102.056 (8), 72.081 (7)	HMDB0004827	[21]
204.123	0.84	Positive	Acetylcarnitine	Level 2	M + H	85.029 (100), 204.124 (77), 60.081 (29), 145.049 (24), 187.018 (5), 122.061 (4), 144.103 (3)	HMDB0000201	[19]
239.092	5.51	Negative	CMPF ^3^	Level 2	M − H	195.103 (100), 239.09 (42), 151.112 (30), 96.961 (17), 238.579 (8), 0 (0), 0 (0)	HMDB0061112	[21]

^1^ Confidence in annotation according to Schymanski system [20]. ^2^ Samples from pregnancy (maternal blood) and delivery (maternal blood and umbilical cord blood) were analyzed and preprocessed simultaneously, resulting in identical identifications. ^3^ 3-carboxy-4-methyl-5-propyl-2-furanpropanoic acid.

## Data Availability

The R-code for conducting the statistical analyses can be obtained from https://gitlab.com/miastravik/dietary-biomarkers (accessed on 26 April 2024). The in-house developed R-based Shiny app (OCEAN) used for metabolite identification can be obtained from https://gitlab.com/parasitetwin/autoannotshiny (accessed on 26 April 2024). Data described in the manuscript will not be made available because it relates to information that could compromise research participant privacy or consent. The food frequency questionnaires are not publicly available due to proprietary rights.

## Supplementary Materials

The following supporting information can be downloaded at https://www.mdpi.com/article/10.3390/metabo14050256/s1, Figure S1: Directed acyclic graph; Figure S2: MSMS spectra matching for choline; Figure S3: MSMS spectra matching for pipecolic acid; Figure S4: MSMS spectra matching for indole-3-lactic acid; Figure S5: MSMS spectra matching for CMPF; Figure S6: MSMS spectra matching for proline betaine; Figure S7: MSMS spectra matching for acetylcarnitine; Figure S8: MSMS spectra matching for lutein; Table S1: Features of interest for MSMS analysis; Supplementary Materials File S1: Regressions and Correlations.
