# Supplementary material for: Biomarker Candidates of Habitual Food Intake in a Swedish Cohort of Pregnant and Lactating Women and Their Infants
Source: Metabolites. 2024 Apr 29;14(5):256. doi: 10.3390/metabo14050256 (PMC11123206; doi:10.3390/metabo14050256)
Supplement: Supplementary file 1 [file metabolites-14-00256-s001.zip › Supplementary Material (Tables & Figures).pdf]

metabolites

Article

Biomarker Candidates of Habitual Food Intake in a Swedish Cohort of Pregnant and Lactating Women and Their Infants

Mia Stråvik [1,*], Olle Hartvigsson [1], Stefania Noerman [1], Anna Sandin [2], Agnes E. Wold [3], Malin Barman [1] and Ann-Sofie Sandberg [1]

[1] Food and Nutrition Science, Department of Life Sciences, Chalmers University of Technology, 412 96 Gothenburg, Sweden; ann-sofie.sandberg@chalmers.se
[2] Pediatrics, Department of Clinical Science, Sunderby Research Unit, Umeå University, 901 87 Umeå, Sweden
[3] Department of Infectious Diseases, Institute of Biomedicine, Sahlgrenska Academy, University of Gothenburg, 413 90 Gothenburg, Sweden
* Correspondence: mia.stravik@chalmers.se

Content

Table S1. Features of interest for MSMS analysis.

Figure S1. Directed acyclic graph.

Figure S1. MSMS spectra matching for choline.

Figure S2. MSMS spectra matching for pipecolic acid.

Figure S3. MSMS spectra matching for indole-3-lactic acid.

Figure S4. MSMS spectra matching for CMPF.

Figure S5. MSMS spectra matching for proline betaine.

Figure S6. MSMS spectra matching for acetylcarnitine.

Figure S7. MSMS spectra matching for lutein.

Table S1. Features of interest for MSMS analysis.

Potential metabolite	mz	rt	Adduct	Mode
**Pregnancy & delivery**				
Anserine	121.070002980776	43.5149993896484	M+2H	Positive
Pipecolic acid	130.084874061977	53.6369667053223	M+H	Positive
Choline	143.071942117219	146.512084960938	M+K	Positive
Proline betaine	144.100677933661	46.4918327331543	M+H	Positive
Pipecolic acid	147.112804007174	35.7742233276367	M+NH4	Positive
Indole-3-acetaldehyde	177.101523417765	55.4220008850098	M+NH4	Positive
Proline betaine	185.127041836108	141.155014038086	M+ACN+H	Positive
Acetylcarnitine	204.122894778965	47.6826438903809	M+H	Positive
Carnosine	227.110415556985	324.526000976562	M+H	Positive
CMPF[1]	241.109775710262	327.502990722656	M+H	Positive
CMPF[1]	263.089872705659	327.503021240234	M+Na	Positive
Lutein	285.22476495678	427.208633422852	M+2H	Positive
3,3'-diindolylmethane	288.15222712711	351.317016601562	M+ACN+H	Positive
Phloretin	307.120055349519	353.700042724609	M+CH3OH+H	Positive
Kaempferol	319.081397751648	363.225006103516	M+CH3OH+H	Positive
Lutein	569.432313465669	468.609008789062	M+H	Positive
Lutein	591.428029828622	408.471984863281	M+Na	Positive
Lutein	601.471061770655	466.823059082031	M+CH3OH+H	Positive
Trimethylamine N-oxide	76.0752816857054	42.3238182067871	M+H	Positive
Ascorbic acid	87.009312096435	52.5139427185059	M-2H	Negative
Ascorbic acid	157.011511330464	35.8087501525879	M-H2O-H	Negative
Proline betaine	178.064127208162	45.9650001525879	M+Cl	Negative
S-methylcysteine	194.046294950205	285.766510009766	M+Hac-H	Negative
Indole-3-lactic acid	204.066708245801	222.788223266602	M-H	Negative
Carnosine	247.082009909926	301.536499023438	M+Na-2H	Negative
Phloretin	333.09181425996	285.901412963867	M+Hac-H	Negative
**4 months postpartum[2]**				
Choline	104.107121986718	40.8259963989258	[M+H]+/[M]+	Positive
Pipecolic acid	130.086400246006	55.4720458984375	[M+H]+	Positive
Proline betaine	144.101857908248	46.318000793457	[M+H]+	Positive
S-methylcysteine	177.070361094278	361.815002441406	[M+H-H2O]+/[M+ACN+H]+	Positive
3-Methyl histidine	192.07457785385	38.1227130889893	[M+H]+/[M+Na]+	Positive
Acetylcarnitine	204.123262821318	50.5887908935547	[M+H]+	Positive
4-hydroxyphenylpyruvate	213.074734423396	37.7863998413086	[M+H]+/[M+CH3OH+H]+	Positive
CMPF[1]	263.08981438261	325.809936523438	[M+H]+/[M+Na]+	Positive
Zeaxanthin	568.426551797752	465.572570800781	[M+H]+/[M]+	Positive
Lutein	569.432313465669	468.609008789062	M+H	Positive
CMPF[1]	239.092153517623	330.2763671875	[M-H]-	Negative

[1] 3-carboxy-4-methyl-5-propyl-2-furanpropanoic acid.

[2] The most probable mz corresponding to a cluster from RamClust in R.

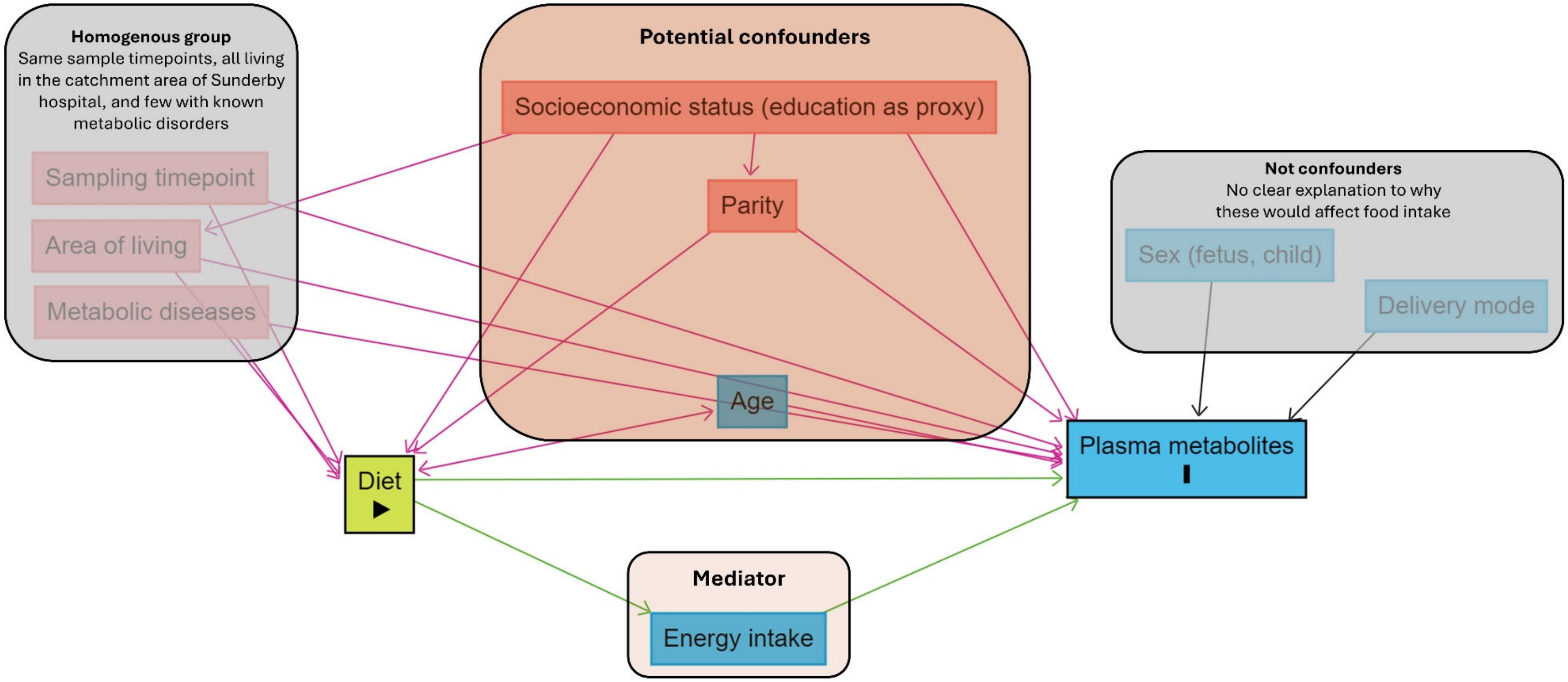

Figure S1. Directed acyclic graph.

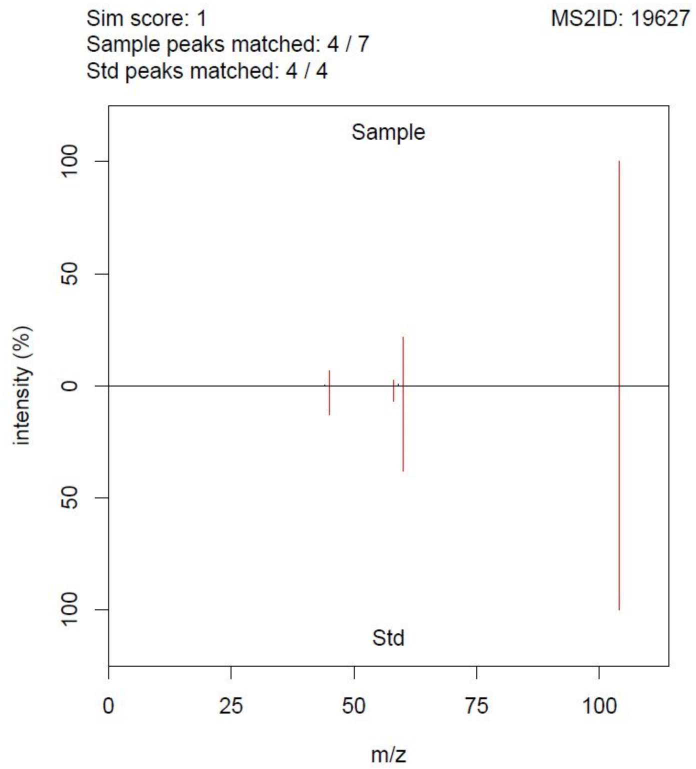

Figure S2. MSMS spectra matching for choline.

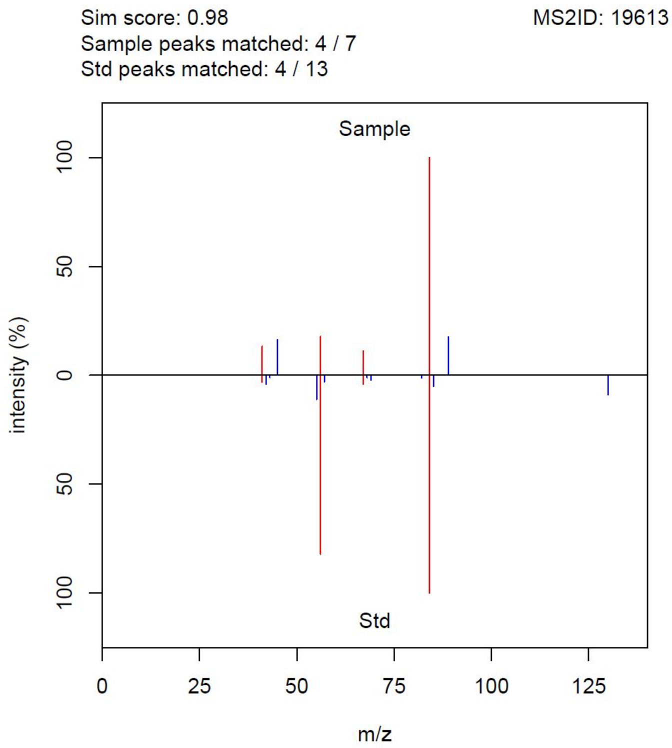

Figure S3. MSMS spectra matching for pipecolic acid.

metabolites

MDPI

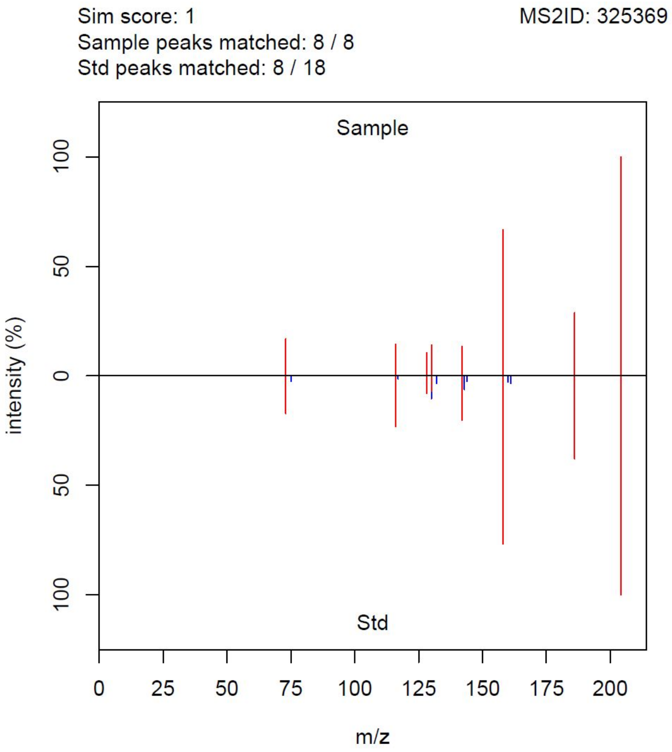

Figure S4. MSMS spectra matching for indole-3-lactic acid.

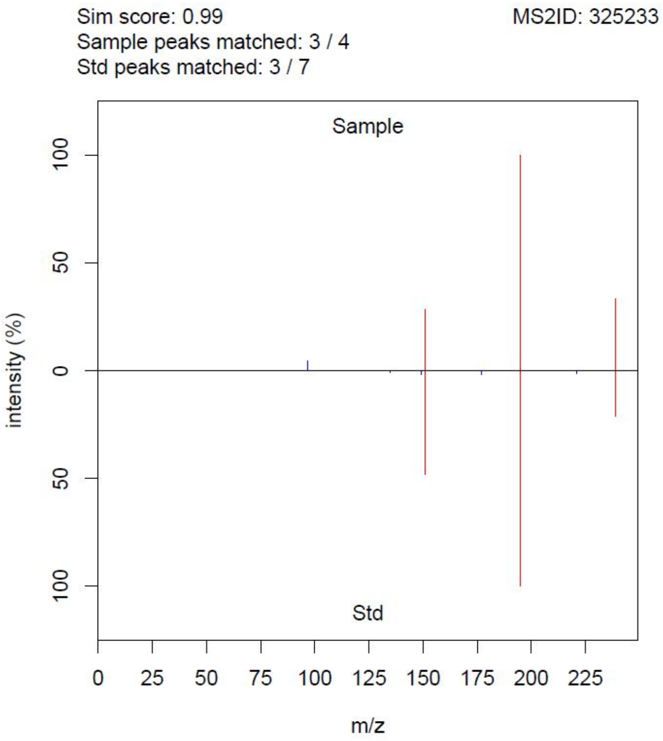

Figure S5. MSMS spectra matching for CMPF.

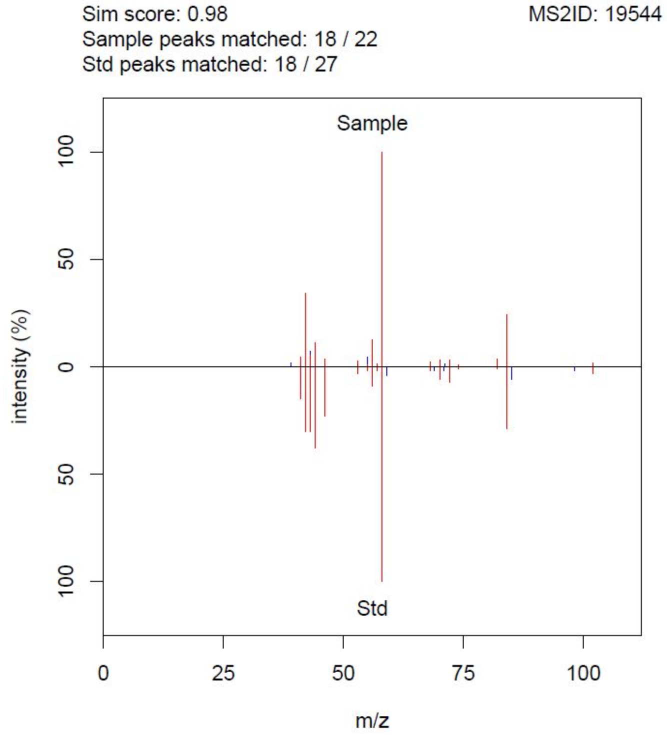

Figure S6. MSMS spectra matching for proline betaine.

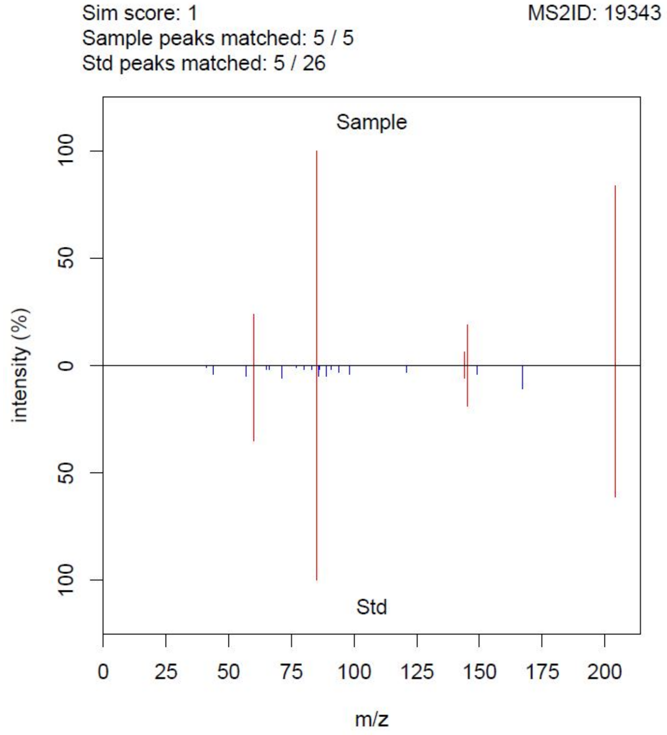

Figure S7. MSMS spectra matching for acetylcarnitine.

metabolites

MDPI

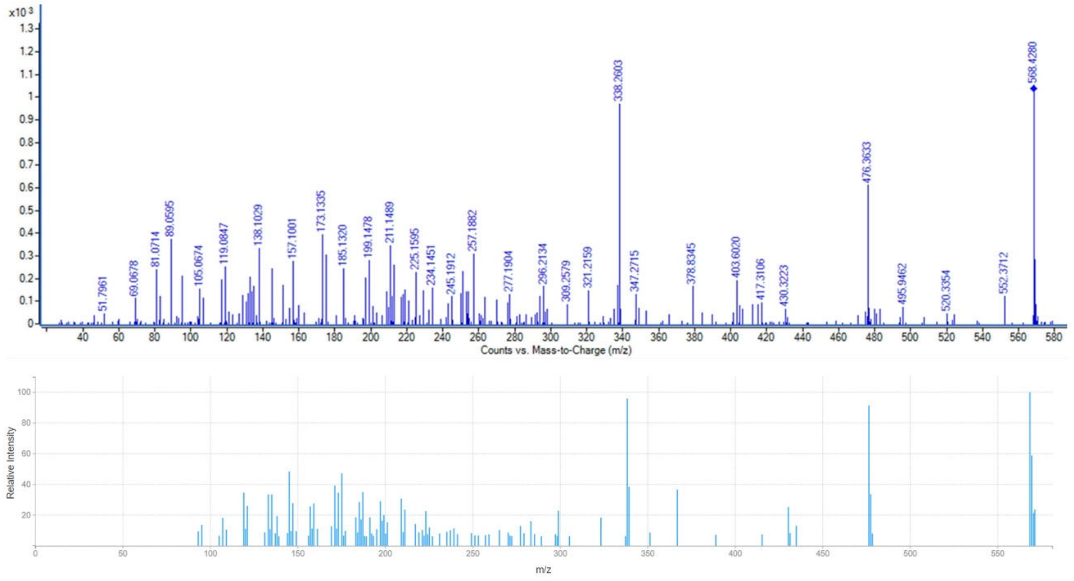

Figure S8. MSMS spectra matching for lutein. Matching was done after a manual check against HMDB spectral library. The reference spectra (bottom image) can be found at: https://hmdb.ca/spectra/ms_ms/2228168.
